# Hysteresis in Heat Capacity of MWCNTs Caused by Interface Behavior

**DOI:** 10.3390/nano12183139

**Published:** 2022-09-10

**Authors:** Nadezhda Bobenko, Valeriy Egorushkin, Alexander Ponomarev

**Affiliations:** Institute of Strength Physics and Materials Science of SB RAS, 2/4 Academichesky Avenue, Tomsk 634021, Russia

**Keywords:** structural disorder, carbon nanotubes, domain structure, heat capacity, temperature hysteresis

## Abstract

The paper is concerned with the study of structural disorder as well as the emergence and causes of heat capacity hysteresis in multiwall carbon nanotubes. The investigation methods are X-ray diffraction analysis, Raman spectroscopy, transmission electron microscopy, and calorimetric tests: thermogravimetric analysis, differential scanning calorimetry, and the thermal relaxation method for heat capacity hysteresis. Multiwall carbon nanotubes are shown to be composed of one or several types of zigzag–armchair domains. The domain structure of nanotube samples is responsible for the generation of uniaxial elastic microstrains and viscoelastic bending strains at domain interfaces. The thermomechanical behavior of interfaces is the chief cause of temperature hysteresis of heat capacity. The number of hystereses corresponds to the number of domain types in the structure, and values of hysteresis are determined by the crystallite size, thermal conductivity, and normal temperature distribution of strain. The found mechanism of heat capacity hysteresis can be helpful in preventing jumps in thermal properties and managing thermal memory in multiwall carbon nanotubes.

## 1. Introduction

Multiwall carbon nanotubes (MWCNTs) feature unique electron, thermal, and mechanical properties, which makes them candidates for use in electronics, chemical, and biological sensors, thermal devices, and energy converters [1,2]. Unique properties of MWCNTs are determined by the material structure [3]. The crystalline structure of MWCNTs directly depends on the method of production and subsequent processing, namely, removal of amorphous carbon, catalyst particles, thermal annealing, etc. [3,4]. 

The carbon nanotube surface is composed of graphene fragments, i.e., crystallites of the zigzag–armchair type. Their size and orientation are the most important characteristics of structural disorder [5,6,7]. The crystallite size La is used as a measure of crystallinity of carbon nanotubes. The quantitative information about La may be received by the three available experimental techniques: Raman spectroscopy, X-ray diffraction, and transmission electron microscopy (TEM) [8]. Raman spectroscopy enables a comparison of the D peak intensity ID (associated with defects and disorder) with the G peak intensity IG (inherent graphite excitation). It was found [6,9] that the ID/IG ratio is proportional to the crystallite size La. X-ray diffraction peaks are used as a measure of interplanar spacing and microstrains in tubes [10]. Transmission electron microscopy is useful in determining the diameter of MWCNTs, and the number and distance between nanotube walls [11,12]. The relation of structural changes to thermal properties is established by calorimetric tests: thermogravimetric analysis (TGA) for the mass change of a sample, differential scanning calorimetry (DSC) for energy dissipation, and the thermal relaxation method for heat capacity hysteresis [13].

Disorder and structural changes in MWCNTs affect the stability of their physical properties and consequently the reliability of devices. In particular, instability in thermal properties (thermal conductivity, heat capacity) can lead to catastrophic failure of devices as a result of overheating or deformation. Instability may present as temperature hysteresis. On the one hand, hysteresis violates the stability of properties and, on the other hand, determines the possibility of thermal memory in materials [14]. The phenomenon of hysteresis is usually associated with phase, structural, magnetic, and other transformations [15,16]. Hysteresis was discovered for heat capacity in nanomaterials [17,18], and in glasses [19]. The reason for hysteresis in fullerenes [17] is the FCC–SC transition. The hysteresis in glass is caused by the structural relaxation and the time of relaxation is determined by the energy barrier, which depends solely on the current value of thermodynamic coordinates [19]. The mechanism of hysteresis in MWCNTs [18] is still unclear.

There also exists a mechanical stress–strain hysteresis, which is governed by structural relaxation of materials [20]. Such hysteresis was observed in MWCNTs [20] and explained by a strain-induced change in the density of samples.

Hysteresis is also influenced by interfaces [21,22,23]. It was found [21] that the applied external stress or strain can effectively adjust the thermal conductivity by changing the density of twin boundaries, thus presenting a peculiar mechanically controlled thermal switch of hysteresis operations. Boundaries of domains can be possible sources of thermal memory [22]. Local strains and stresses and their relation to bending of carbon nanotubes and relaxation were studied in situ [23].

It was shown [24,25,26] that low-temperature synthesis, hexagonal strain, and residual internal stresses cause a zigzag–armchair domain structure to form in tubes due to a thermodynamic phase transition. Another factor affecting the physical properties of tubes is the crystallite size, i.e., intercrystalline interfaces. 

This work is devoted to the study of thermomechanical and relaxation behavior of domain interfaces and their role in the low-temperature hysteresis of heat capacity.

## 2. Materials and Methods

Two types of MWCNTs are used in experiments: sample series S1 with the average outer diameter Ø18 nm and sample series S2 with Ø7.2 nm. The average length of MWCNTs is 30 μm, and the density is ~2 g/cm^3^.

Multiwall carbon nanotubes were synthesized using the chemical vapor deposition (CVD) method by decomposing ethylene on the bimetallic catalyst at 670 °C [26]. Sample S1 was obtained using 40% Fe–Co/CaCO_3_ catalysts, and S2–40% Fe–Co/Al_2_O_3_ [27].

The MWCNT powder was treated with 15% HCl and then washed with distilled water to neutral pH. This procedure and subsequent drying in air reduced the concentration of impurities in MWCNTs. The used catalyst composition provides MWCNTs with a small number of defects and a low amount of inorganic impurities. The concentration of C, Fe, Co, O, and Cl is measured by energy dispersive X-ray spectroscopy (EDS). Carbon amounts to ~99 wt%, and the other elements are near 0.3–0.5 wt% [18]. 

The structure, chemical composition, and thermal properties of MWCNTs are studied using transmission electron microscopy (TEM), X-ray diffraction, Raman spectroscopy, thermogravimetric analysis, differential scanning calorimetry, and the thermal relaxation method.

Structural investigations are performed on a JEOL JEM-2200FS (JEOL Ltd., Tokyo, Japan) transmission electron microscope in the bright-field mode at the accelerating voltage 200 kV. Data on the diameter and interplanar spacing of nanotubes are obtained using the licensed ImageJ 1.53r software incorporated into the TEM software package.

The crystalline structure of MWCNTs is studied by the X-ray diffraction analysis method (XRD, Malvern Instruments, Malvern, UK) using a PANalytical Empyrean diffractometer (CuKα1, λp = 1.54056 Å) with Bragg–Brentano θ–2θ geometry. The derived crystallographic structures are identified from the Crystallography Open Database (COD). 

The structure imperfection of carbon nanotubes is estimated using Raman spectroscopy. This investigation is conducted at the Scientific Park of NR Tomsk Polytechnic University using an NT-MDT-Solar AFM/Raman spectrometer (NT-MDT, Moscow, Russia) at the laser radiation wavelength 633 nm and 100× magnification. Raman G and D bands are analyzed by Lorentzian functions using the XPSPEAK 4.1 software. The size of crystallites in samples is estimated in terms of peak intensities ID and IG by the formula $L_{a}=\left(2.4\cdot 10^{-10}\right)\lambda^{4}{\left(\frac{I_{D}}{I_{G}}\right)}^{-1}$ [9].

Thermogravimetric analysis and differential scanning calorimetry are used to study temperature changes in the mass of the material and energy dissipation during its heating, thus establishing the causes of dissipation, total content of carbon, its structural modifications, oxygen-containing groups, and residual metal catalyst. Thermal tests on MWCNTs are carried out using an STA 449 F3 Jupiter synchronous thermal analyzer (Netzsch, Selb, Germany) combined with a QMS 403CAeolos mass spectrometer (Netzsch, Germany) in oxygen-free argon. Samples S1 and S2 weighing 3.4 and 3.2 mg, respectively, are heated from 40 to 1200 °C at the heating rate 10 °C/min in a dynamic argon atmosphere (50 mL/min).

Low-temperature heat capacity is investigated on solid tablets compacted from MWCNT powder under the pressure 1.1 GPa. Samples about 2–3 mm in thickness and 2–6 mg in weight are cut from the tablets with the diameter 10 mm. The specific heat of MWCNTs is measured [18] in the temperature range from 1.8 to 275 K by the thermal relaxation method using a Quantum Design physical property measurement system (PPMS, San Diego, CA, USA). The thermal contact between a sample and the PPMS platform is improved by the use of Apiezon grease. Temperature is measured by a Cernox resistance thermometer (Lake Shore Cryotronics, Inc., Westerville, OH, USA).

Theoretical studies are performed within the thermodynamics of structural transformations, nonequilibrium thermodynamics, balance equation, Landau–Khalatnikov equation, and probability theory.

## 3. Experimental Results and Discussion

### 3.1. Transmission Electron Microscopy

**Figure 1:**
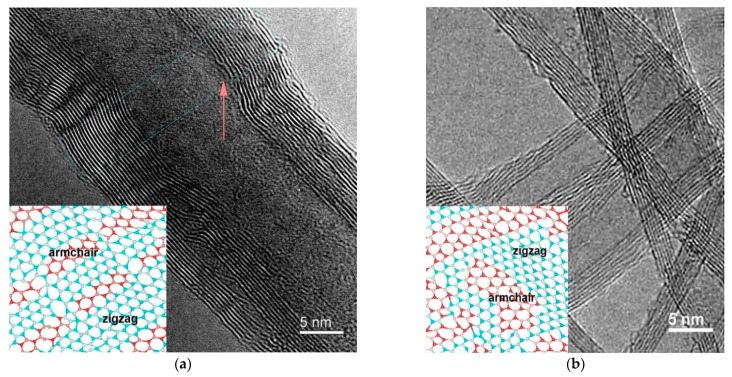
High-resolution TEM images of samples S1 (**a**) and S2 (**b**).

Figure 1 shows TEM images of samples S1 and S2. The TEM image for S1 in Figure 1 is taken from [24] for the necessary comparison of the two samples’ structures. Both samples are seen to have a domain structure. In sample S1, the boundaries of zigzag–armchair domains are almost parallel to each other, as illustrated in the inset of Figure 1a. Domains of sample S2 are distributed in a mosaic manner (see the inset in Figure 1b). These insets illustrate different modes structures. Zigzag–armchair domains in MWCNTs in TEM [28] are connected with broken and curved of the tube walls. These walls separate islands of mosaic graphene zigzag and armchair structures. The STM studies lead to the same results [29,30]. The transverse profiles from the nanotube axis to its outer layer show that the average interlayer spacing is ~0.349 nm for S1 and ~0.352 nm for S2, and the average outer diameter of the tubes is 18 nm (20 walls) and 7.2 nm (10 walls), respectively.

### 3.2. X-ray Diffraction

**Figure 2:**
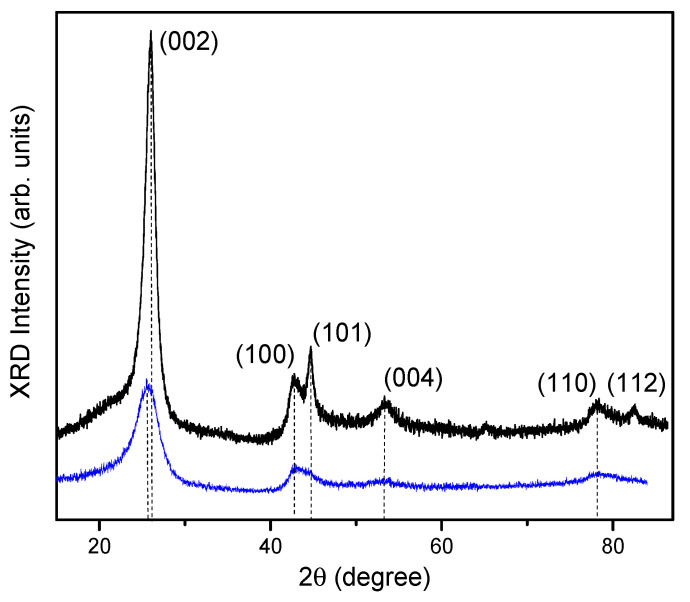
X-ray diffraction patterns for samples S1 (black curve) and S2 (blue curve).

X-ray diffraction patterns of samples S1 and S2 are plotted in Figure 2. X-ray diffraction patterns for S1 are shown in Figure 2 from [24] to clearly show the differences for samples with different structures. Angular positions of the main peaks, their relative intensities, full width at half maximum (FWHM) of an X-ray diffraction, etc., derived from the X-ray patterns are given in Table 1.

Dwell on the X-ray diffraction patterns of samples S1 and S2. Diffraction peaks (002) at 2θ ≈ 26°, (100) at 2θ ≈ 42.8°, (101) at 2θ ≈ 44.7°, and (110) at 2θ ≈ 78.4° correspond to the hexagonal structure of graphite according to COD 96-901-2231. A peak near 2θ ≈ 65° in sample S2 corresponds to the Fe catalyst residues according to COD 96-411-3932. Peak (112) at 2θ ≈ 81.2° corresponding to the (hkl) plane is present in S1 and absent in S2, which points to a more perfect structure of S1 [31]. All peaks in the X-ray diffraction patterns of samples S1 and S2 are blurred. In this case, broadening is larger in S2, especially for the (002) and (110) peaks.

Maximum peaks in samples S1 and S2 are peaks (002). The asymmetry of these peaks indicates the presence of domains (different crystal structures) in MWCNTs [32]. Peaks (101) are approximately of the same intensity in S1 and S2. Peak (100) in S1 has a significantly lower intensity (Table 1). This means that the crystallinity disruption (zigzag–armchair structure) in S1 is oriented predominantly along axis Z [32]. In sample S2, the (101) and (100) peaks are close in intensity, which is characteristic of the coexistence of zigzag and armchair structures [32]. The coincidence of 2θ of peaks (100) in both samples indicates the predominance of zigzag structures in them [33]. This is also confirmed by the presence of peak (112) in tubes S1. The lack of such a peak in S2 points to the existence of zigzag and armchair structures [32].

The presence and splitting of the (004) peak in sample S1 indicates the differences in interlayer spacing along the tube axis with every next layer, which is associated with a possible single-domain structure (bamboo-like one) [34]. In multidomain structures, the interlayer spacing is constant [34]. This peak is missing in S2 [31]. The average interplanar spacing calculated by the standard formula is smaller in sample S1: d_002_ = 3.43 Å and d_100_ = 2.10 Å in S1; d_002_ = 3.49 Å and d_100_ = 2.11 Å in S2 (Table 1). This agrees with the experimental data [35] and our TEM measurements. In addition, there is an increase in d_002_ and a decrease in d_100_ from the values for graphite (d_002_ = 3.35 Å, d_100_ = 2.46 Å) [36].

Microstrain (ε) is calculated by the standard formula $\varepsilon_{\left(\mathrm{hkl}\right)}=\frac{{\Delta B}_{\left(\mathrm{hkl}\right)}}{4\mathrm{tg}\left(\theta_{\left(\mathrm{hkl}\right)}\right)}$ [37], where ΔB is the FWHM, and θ is the diffraction angle. The calculated values of $\varepsilon_{\left(\mathrm{hkl}\right)}$ for all peaks are given in Table 1.

The maximum strain in S1 and S2 is ε_zz_, which is calculated in terms of the half-width of the 002 peak and corresponds to a higher level of interlayer deformation. Along with ε_zz_, sample S2 is characterized by significant ε_xx_ (peak (100)) corresponding to deformation at the zigzag–armchair boundary along the X-axis. Sample S2 also experiences ε_yy_ close in value to ε_xx_. The presence of ε_zz_, ε_xx_, and ε_yy_ in sample S2 points to a multimode domain structure [24]. The only strain ε_zz_ found in S1 indicates a predominantly single-mode structure. At high tensile–compressive strains, inhomogeneous domain boundaries experience bending deformation [38]. Bending can be induced by different stresses at different points of a boundary [38].

The size of coherent scattering regions (CSR) is calculated by broadening of the (002) peak according to the formula $D=\frac{\lambda_{p}}{\Delta \mathrm{Bcos}\theta }$ [37], where D is the CSR size, λ_p_ is the X-ray wavelength, ΔB is the FWHM of X-ray diffraction peak (110), and θ is the corresponding diffraction angle. The calculations show that the CSR size in S1 is not larger than 2.3 times that of sample S2.

### 3.3. Raman

**Figure 3:**
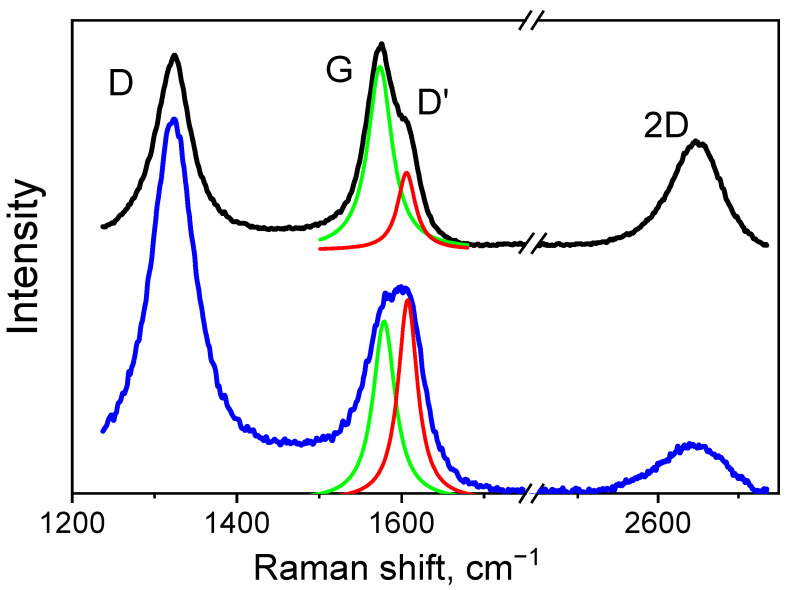
Raman spectra of samples S1 (black curve) and S2 (blue curve). Decomposition of the G peak into Lorentzian spectra (green and red curves).

Raman spectra for S1 and S2 in the range 1200–2800 cm^−1^ are exhibited in Figure 3. All Raman spectra are normalized to the G-mode intensity. The minimum intensity in the range is set to zero. The excitation wavelength λ = 633 nm. The Raman spectra of both samples include peaks D, G, D’, and 2D. The main characteristics of these spectra are cited in Table 2.

The decomposition of the G peak into Lorentzian spectra shows that it splits into two: the G and D’ peaks for both samples. Positions of the G and D’ maxima for S1 and S2 are shown in Table 2. The peak near 1575 cm^−1^ for sample S1 and the peak near 1580 cm^−1^ for sample S2 give an indication of the presence of a zigzag structure. The peaks near 1593 cm^−1^ and 1599 cm^−1^ imply the existence of an armchair structure [39]. In sample S1, the relative peak intensity I_G_/I_D’_ is 2.5, which suggests the predominance of a zigzag structure. In sample S2, these peaks are close in intensity, which is evidence for the existence of both structures [39].

Splitting of the G peak and redshift of the D peak in both samples is indicative of the presence of uniaxial deformation in the material [40]. In sample S1, the G peak shift is larger than that in S2, which is suggestive of higher strain ε_zz_ [40] and agrees with our X-ray measurements (Table 1).

Different intensities of the D mode for samples S1 and S2, in the absence of concentration defects, are associated with their different structures. A high intensity of the D peak corresponds to a large number of zigzag–armchair boundaries [41,42].

In addition, Raman spectra of both samples have a 2D band at 2647 cm^−1^, which also shows the presence of structural disorder [6]. Structural elements of this disorder are crystallites, whose calculated size $L_{a}$ is given in Table 2. The crystallite size $L_{a}$ in samples S1 is not larger than 2.2 times that in samples S2, which is due to the single-mode domain structure of S1 as compared to the multimode structure in S2. The ratio of the CSR sizes (XRD) in these samples is also 2.3 (Table 1).

### 3.4. Thermogravimetric Analysis and Differential Scanning Calorimetry 

**Figure 4:**
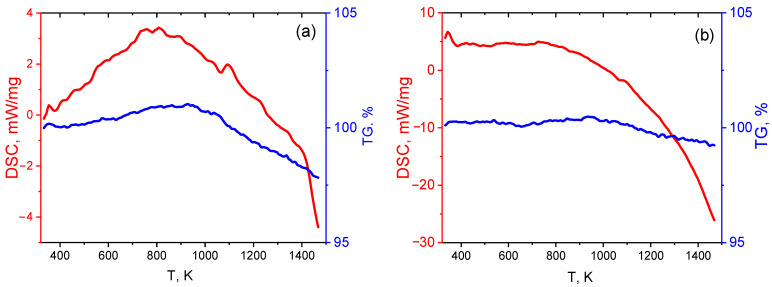
DSC (red) and TGA (blue) curves for samples S1 (**a**) and S2 (**b**).

In S1, the dissipated energy is about five times less than that in S2 (Figure 4). At the same time, the constant mass of the samples at temperatures below 600 K indicates internal sources of nonequilibrium, i.e., microstrains (microstresses).

At temperatures above 700 K, the energy drop (release) begins in S2. The corresponding ~1% reduction in mass is observed in the temperature range 950–1500 K. This may be related to the fact that, at 700–950 K, oxygen does not release but forms CO_2_. When the temperature reaches ~950 K and the isobaric potential of CO becomes smaller, CO gas formation and release occur [13]. For sample S1, the decrease in energy (by a factor of 5) in the temperature range 700–1500 K and in mass (by ~2%) at 900–1500 K has the same reason.

The small change in mass suggests a low content of oxygen in the samples, the absence of other allotropic forms of carbon, and catalyst particles. The variation in dissipated energy at the constant mass at temperatures up to 600 K points to a significant role of the internal structure. The strongly different temperature behavior of the DSC curves for S1 and S2 is evidence for different internal structures.

The DSC/TGA data show no oxidation of samples S1 and S2 at temperatures below 500–600 K. However, energy dissipation is observed at these temperatures.

### 3.5. Specific Heat

Temperature dependences C(T) for S1 and S2 were previously derived [18]. The values of C(T) in S1 are ~10% lower than that in S2 in the entire temperature range. For both samples, C(T)~T^3^ up to the temperature 5 K, and C(T)~T^2^ from 5 to 35 K. In the temperature range from ~35 to 110 K, the temperature dependence C(T)~T^1.5^ for S1, and C(T)~T for S2. Above 110 K, C(T)~T for both samples. The same behavior is typical of other MWCNTs [43,44,45,46].

The measurement of heat capacity during cyclic heating and cooling revealed the phenomenon of hysteresis in samples S1 and S2 [18]. Hysteresis occurs in the temperature range 35–70 K for S1 and 35–110 K for S2. 

The temperature behavior of heat capacity varies due to the change in the dispersion law of the bending mode $\omega_{k}$ [18]. Its variation in structurally disordered samples can be caused by phonon scattering by crystallite boundaries [24]. The mechanism of hysteresis in such materials can be also related to the presence of crystallites. 

**Figure 5:**
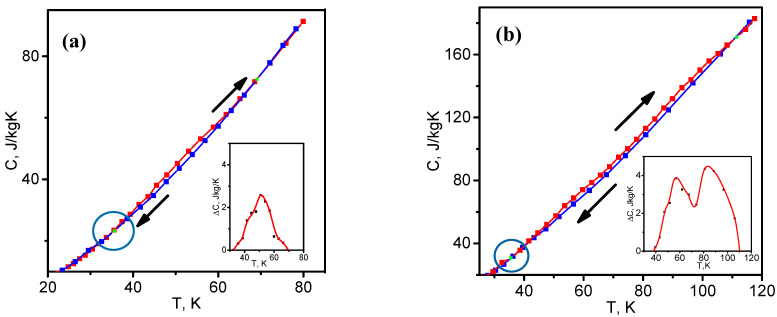
Heat capacity of samples S1 (**a**) and S2 (**b**) in heating (red curves) and cooling (blue curves). Hysteresis of heat capacity ΔC(T) is given in the insets, and a region near the hysteresis onset temperature during the heating–cooling process is circled.

For a detailed analysis of the hysteresis mechanism, we build temperature dependences C(T) and ΔC(T) during the heating–cooling process based on the experimental data [18] in the temperature range 25–120 K (Figure 5).

From Figure 5a, it is seen that sample S1 has a hysteresis in the temperature range 35–70 K, with the maximum ΔC=2.6 J/kgK at T = 53 K. Sample S2 (Figure 5b) is characterized by a hysteresis with two maxima (${\Delta C}_{1}=4\;J/\mathrm{kgK}$ at T = 57 K and ${\Delta C}_{2}=4.5\;J/\mathrm{kgK}$ at T = 90 K) in the temperature range 35–110 K. The coincidence of the temperature ranges 35–70 K and the temperature of the maxima for S1 and S2 (the first hysteresis) points to the fact that these two phenomena have the same cause. The two hystereses of sample S2 are found in the temperature ranges 35–70 and 70–110 K (Figure 5b, inset). The second hysteresis in S2 can be evidently traced to another cause. The value of the first hysteresis in sample S2 is almost twice as much as that in sample S1. This means that sample S2 contains a larger number of hysteresis sources similar to those in S1.

The structural experiments conducted on samples S1 and S2 proved the presence of structural disorder and the absence of concentration disorder. Structural disorder is due to a single-mode domain structure in S1 and a multimode domain structure in S2. The formation of domains is associated with equilibrium microstrains: ε_zz_ in S1, and ε_zz_, ε_xx_ in S2. The calorimetric studies showed that samples S1 and S2 have not only equilibrium but also nonequilibrium microstrains, which can determine the appearance of heat capacity hysteresis.

In search of the causes and mechanism of temperature hysteresis in C(T), we turn to [24,25,26]. 

## 4. Energy Dissipation and Temperature Hysteresis of Heat Capacity 

It was shown [24,25,26] that synthesis conditions of MWCNTs significantly affect their structure and lead to the accumulation of macrostrains and stresses. As a result, thermodynamic equilibrium domains of various hexagonal structures appear in nanotubes. The formation of a domain structure during the MWCNT manufacture depends on the internal elastic tensile–compressive stresses $\sigma_{\mathrm{ii}}$ (i = x,z). Thus, under $\sigma_{\mathrm{zz}}<0$, a single-mode domain structure of the bamboo-like type is formed. Other stresses induce a multimode structure of the mosaic type.

Gibbs energy for the single-mode structure has the form [24]

(1) $$F^{\left(1\right)}=\frac{2}{C_{2}}\left(a_{2}+\frac{2a_{1}C_{1}}{C_{2}}\right)\varepsilon_{\mathrm{zz}}^{2}-\frac{a_{2}}{C_{2}}\sigma_{\mathrm{zz}}\varepsilon_{\mathrm{zz}}$$

For the multimode structure, Equation (1) is added with

(2) $$F^{\left(2\right)}=\frac{4}{C_{1}}\left(a_{1}+\frac{a_{2}C_{2}}{C_{1}}\right)\varepsilon_{\mathrm{xx}}^{2}-\frac{a_{2}}{C_{1}}\sigma_{\mathrm{xx}}\varepsilon_{\mathrm{xx}}$$

where $\varepsilon_{\mathrm{ii}}$ and $\sigma_{\mathrm{ii}}$ are the equilibrium strains and stresses, and the coefficients $a_{1}$, $a_{2}$, $C_{1}$, $C_{2}$ are defined in [24]. The experimental values of $\varepsilon_{\mathrm{ii}}$ found from the X-ray diffraction patterns are given in Table 1 and discussed above.

However, as follows from the TGA tests, samples S1 and S2 have, apart from equilibrium $\varepsilon_{\mathrm{ii}}$, thermodynamic nonequilibrium strains at low temperatures which cause energy dissipation (Figure 4). Such strains and Gibb’s energy determined in (1) and (2) are related by the Landau–Khalatnikov equation [47]:

(3) $$\frac{{d\varepsilon }_{\mathrm{ii}}}{\mathrm{dt}}=-\gamma_{i}\frac{{\mathrm{dF}}^{\left(i\right)}}{{d\varepsilon }_{\mathrm{ii}}},$$

where $\gamma_{i}$ is the kinetic coefficient, and $\frac{{\mathrm{dF}}^{\left(i\right)}}{{d\varepsilon }_{\mathrm{ii}}}$ is the internal friction force.

Let us calculate the derivative in (3). With (1) and (2), we obtain

(4) $$\frac{{d\varepsilon }_{\mathrm{ii}}}{\mathrm{dt}}=\frac{\sigma_{\mathrm{ii}}}{\eta_{i}},$$

where $\eta_{z}=\gamma_{z}\left(\frac{a_{2}}{C_{2}}-\frac{4}{C_{2}E}\left(a_{1}C_{1}+\frac{a_{2}C_{2}}{2}\right)\right)$ and $\eta_{x}=\gamma_{x}\left(\frac{a_{2}}{C_{1}}-\frac{4}{C_{1}E}\left(a_{1}C_{1}+a_{2}C_{2}\right)\right)$ are the effective viscosity coefficients, and E is Young’s modulus. From (1)–(4), it follows that an energy dissipated at low temperatures is elastic energy, and dissipation occurs due to effective viscosity in the medium.

Under external stresses, such behavior could induce a mechanical hysteresis [15,16], but, since internal stresses do not work, the elastic energy is dissipated into heat.

Thus, in addition to equilibrium strains and stresses that form the MWCNT domain structure, there are also many nonequilibrium internal strains that do not perform mechanical work but only dissipate into heat. The balance equation for energy dissipation E can be written as [47]

(5) $$\rho \frac{\mathrm{dE}}{\mathrm{dT}}=\kappa \mathrm{grad}\;\mathrm{divT}$$

where ρ is the material density, κ is thermal conductivity, and T is the temperature. 

As sample S1 has a single-mode structure with $\varepsilon_{\mathrm{zz}}$, sample S2 has multimode domain structures with $\varepsilon_{\mathrm{zz}}$ and $\varepsilon_{\mathrm{xx}}$, and the heat propagates over the tube surface, Equation (5) can be divided into independent one-dimensional equations:

(6) $$\frac{\mathrm{dE}}{\mathrm{dt}}=\frac{\kappa }{\rho }\frac{d}{{\mathrm{dx}}_{i}}\left(\frac{\mathrm{dT}}{{\mathrm{dx}}_{i}}\right)$$

The left-hand side of (6) is represented as

(7) $$\frac{\mathrm{dE}}{\mathrm{dt}}=\frac{\mathrm{dE}}{\mathrm{dT}}\frac{1}{\frac{{d\varepsilon }_{\mathrm{ii}}}{\mathrm{dT}}}\frac{{d\varepsilon }_{\mathrm{ii}}}{\mathrm{dt}}$$

Here, $\frac{\mathrm{dE}}{\mathrm{dT}}=\Delta C$ is the hysteretic contribution of the dissipated energy to the heat capacity. A simple rearrangement of Equations (6) and (7) gives

(8) $${\Delta C}_{i}=\frac{{\kappa \;\eta }_{i}}{\rho_{i}\sigma_{\mathrm{ii}}L_{a}^{i}}\frac{{d\varepsilon }_{\mathrm{ii}}}{{\mathrm{dx}}_{i}}$$

From (8), it follows that ΔC is determined by inhomogeneous nonequilibrium strain $\varepsilon_{\mathrm{ii}}$, which results in bending of interfaces [38]. In addition, ΔC depends on the density of interfaces (crystallite sizes), the density and thermal conductivity of the material, as well as on the effective viscosity and internal stresses.

The temperature behavior of ΔC is governed by the temperature distribution of interfacial strains $\varepsilon_{\mathrm{ii}}$. Since the $\varepsilon_{\mathrm{ii}}$ value is the sum of numerous loosely coupled random local strains, each of which makes a small contribution to $\varepsilon_{\mathrm{ii}}$, then the distribution $\varepsilon_{\mathrm{ii}}\left(T\right)$ is a normal distribution according to the central limit theorem of probability theory [48]. Therefore, the experimental dependence ΔC(T) is approximated to the Gaussian distribution, and the physical quantities of (8) and ${\Delta C}_{i}$ are estimated by comparing with the approximation parameters.

**Figure 6:**
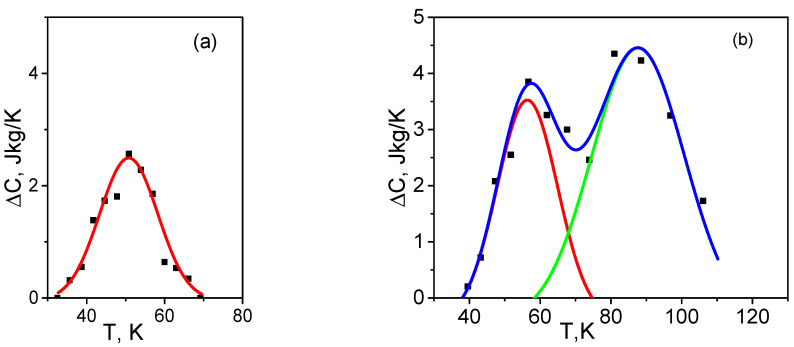
Experimental dependence ΔC(T) (solid squares) and its Gaussian approximation of samples S1 (**a**) and S2 (**b**).

The dependence ΔC(T) approximated using the Gaussian function $f\left(t\right)=\mathrm{Aexp}\left(-\frac{2{\left(T-T_{\max}\right)}^{2}}{\alpha^{2}}\right)$ and the experimental hysteresis values are shown in Figure 6. Here, A is the peak amplitude, T_max_ is the maximum temperature, and α is the dispersion.

Approximation is performed for the hysteresis induced by microstrain ε_zz_ in sample S1 and for the hysteresis associated with two microstrains ε_zz_ (red curve) and ε_xx_ (green curve) in sample S2. The temperature dependence of the total hysteresis in sample S2 is also plotted (blue curve). The approximation parameters are cited in Table 3. The parameters for the first and second hysteresis in sample S2 are given through a slash.

As $\eta_{i}\frac{{d\varepsilon }_{\mathrm{ii}}}{{\mathrm{dx}}_{i}}$ ≈$\frac{\sigma_{\mathrm{ii}}}{c}$ (c ~ 104 m/s is the average speed of sound), the maximum ${\Delta C}_{\max}$ can be finally written in the form

(9) $${\Delta C}_{\max}=\frac{\kappa \;}{{c\rho L}_{a}^{i}}$$

This means that the hysteresis value varies in different samples by changing only the crystallite length L_a_ and thermal conductivity. The ${\Delta C}_{\max}$ values are also given in Table 3. They are in agreement with the experimental values (Figure 5): ${\Delta C}_{\max}=2.6\;J/\mathrm{kgK}$ for sample S1, ${\Delta C}_{\max}{}_{1}=4\;J/\mathrm{kgK}$ and ${\Delta C}_{\max2}=4.5\;J/\mathrm{kgK}$ for sample S2.

A similar effect of domain interfaces on thermal properties was found in [21,51]. 

## 5. Conclusions

In this work, we found the cause and mechanism of the temperature hysteresis of heat capacity, which is unrelated to phase and other transformations. Hysteresis is caused by the formation of domain and crystallite interfaces. Nonequilibrium bending of these interfaces can initiate heat flow and its dissipation, resulting in the hysteresis of heat capacity. The number of hystereses corresponds to the number of different types of interfaces (domain structures), and the hysteresis value is determined by the density of these interfaces.

The microscopic mechanism of dissipation and its temperature range are associated with phonon scattering by crystallite interfaces and nontrivial effects of phonon hybridization. However, this is the subject of further investigation.

## Figures and Tables

**Table 1:** X-ray characteristics.

Sample	2θ	hkl	Intensity	FWHM	Interplanar Spacing d, Å	CSR Size (D), nm	Microstrain, <ε>
S1	25.953	002	100	1.4679	3.43	7	0.0649
	42.84	100	28.83	1.0861	2.10		0.0126
	44.71	101	34.96	1.2711			-
	78.38	110	24.3	1.7938			0.0175
	81.19	112	21.03	-			0.0065
S2	25.531	002	100	2.9733	3.49	3	0.0534
	42.84	100	36.72	1.2917	2.11		0.0179
	44.71	101	33.66	1.9842			-
	78.39	110	32.9	5.7241			0.0157

**Table 2:** Characteristics of Raman spectra of MWCNTs.

Sample	D (cm^−1^)	G (cm^−1^)	D’ (cm^−1^)	2D (cm^−1^)	I_D_/I_G_	L_a_ (nm)
S1	1323	1575	1606	2647	0.94	41
S2	1322	1580	1607	2647	1.78	19

**Table 3:** Physical quantities governing the dependence ΔC(T).

	**S1**	**S2**
A (J/kgK)	2.5	3.9/4.9
T_max_ (K)	51	56/88
α (K)	15	17/26
κ (W/mK)	1.12 [49]	1.44 [50]
ρ (kg/m^3^)	2·10^3^	2·10^3^
L_a_ (nm)	41	19
ΔC_max_ (J/kgK)	2.7	3.8/6

## Data Availability

The data presented in this study are available on request from the corresponding author.
